# Effect of spinal anesthesia-induced deafferentation on pain processing in healthy male volunteers: A task-related fMRI study

**DOI:** 10.3389/fpain.2022.1001148

**Published:** 2022-11-30

**Authors:** Elske Sitsen, Najmeh Khalili-Mahani, Mischa de Rover, Albert Dahan, Marieke Niesters

**Affiliations:** ^1^ Department of Anesthesiology, Leiden University Medical Center, Leiden, Netherlands; ^2^ McGill Centre for Integrative Neuroscience, Montreal Neurological Institute, McGill University, Montreal, QC, Canada; ^3^ Department of Clinical Psychology, Institute of Psychology, Leiden University, Leiden, Netherlands; ^4^ Leiden Institute of Brain and Cognition, Leiden, Netherlands

**Keywords:** deafferentation, pain, spinal anesthesia, task-related fMRI, sensorimotor cortex, inferior parietal lobule, randomized controlled trial, pain perception

## Abstract

**Background:**

Spinal anesthesia causes short-term deafferentation and alters the crosstalk among brain regions involved in pain perception and pain modulation. In the current study, we examined the effect of spinal anesthesia on pain response to noxious thermal stimuli in non-deafferented skin areas using a functional magnetic resonance imaging (fMRI) paradigm.

**Methods:**

Twenty-two healthy subjects participated in the study. We performed a task-based fMRI study using a randomized crossover design. Subjects were scanned under two conditions (spinal anesthesia or control) at two-time points: before and after spinal anesthesia. Spinal anesthesia resulted in sensory loss up to dermatome Th6. Calibrated heat-pain stimuli were administered to the right forearm (C8-Th1) using a box-car design (blocks of 10s on/25s off) during MRI scanning. Pain perception was measured using a visual analogue scale (1–100) at the beginning and the end of each session. Generalized estimating equations were used to examine the effect of intervention by time by order on pain scores. Similarly, higher-level effects were tested with appropriate general linear models (accounting for within-subject variations in session and time) to examine: (1) Differences in BOLD response to pain stimulus under spinal anesthesia versus control; and (2) Effects of spinal anesthesia on pain-related modulation of the cerebral activation.

**Results:**

Complete fMRI data was available for eighteen participants. Spinal anesthesia was associated with moderate pain score increase. Significant differences in brain response to noxious thermal stimuli were present in comparison of spinal versus control condition (post—pre). Spinal condition was associated with higher BOLD signal in the bilateral inferior parietal lobule and lower BOLD signal in bilateral postcentral and precentral gyrus. Within the angular regions, we observed a positive correlation between pain scores and BOLD signal. These observations were independent from order effect (whether the spinal anesthesia was administered in the first or the second visit). However, we did observe order effect on brain regions including medial prefrontal regions, possibly related to anticipation of the experience of spinal anesthesia.

**Conclusions:**

The loss of sensory and motor activity caused by spinal anesthesia has a significant impact on brain regions involved in the sensorimotor and cognitive processing of noxious heat pain stimuli. Our results indicate that the anticipation or experience of a strong somatosensory response to the spinal intervention might confound and contribute to increased sensitivity to cognitive pain processing. Future studies must account for individual differences in subjective experience of pain sensation within the experimental context.

## Introduction

Spinal anesthesia is induced by injection of a controlled dose of local anesthetics in the cauda equina, resulting in temporary and localized loss of sensation in the lower part of the body. Spinal anesthesia causes a transient pharmacological deafferentation of the peripheral nervous system by blocking the sodium channels of the nerves, thus inhibiting the afferent and efferent signaling between the peripheral and central nervous system that process sensory-motor and pain inputs.

Spinal anesthesia provides a plausible experimental model for studying pain associated with peripheral nerve damage. For example, phantom limb pain is believed to be related to alterations in the somatotopic map in the primary sensory and motor cortex resulting from loss of peripheral signaling from nerves of the affected limb to the central nervous system (1). We have used this experimental model in a previous resting state fMRI study (RSfMRI), to show that indeed a pharmacological spinal deafferentation was associated with increased pain scores (hyperalgesia) at the non-deafferented skin areas. Moreover, we observed changes in connectivity between specific brain areas and several canonical resting state networks (2). Namely, increased pain sensation was correlated with changes in functional connectivity of the thalamus to the thalamo-prefrontal network, and changes in functional connectivity of the anterior cingulate cortex and insula to the thalamo-parietal network (2).

A limitation of the previous RSfMRI study was that the noxious stimuli were administered before or after the scan, as such we could not make inferences about the impact of spinal anesthesia on CNS processing of pain stimuli. Furthermore, our previous RSfMRI results were contingent on spontaneous fluctuations within specific canonical networks of interest. The aim of the current study was to overcome those limitations by investigating differences caused by spinal anesthesia to brain activation (BOLD response to a calibrated, timed, pain task).

In a randomized cross-over task-based fMRI study we examined whether spinal anesthesia modulated pain perception in the non-deafferentated skin areas and whether spinal anesthesia changed the brains response to pain stimuli and if those changes were associated with variation in pain perception due to spinal anesthesia.

## Materials and methods

### Study design

The study had a randomized crossover design and involved two visits (spinal anesthesia session or control session), at least one week apart. Randomization of the order of the two visits was performed using a computer-generated randomization list. Because the spinal anesthesia causes a strong reaction (temporary paralysis of the legs), the study was unable to be conducted blindly. Because the Ethics committee did not give permission to administer a sham injection, we performed two fMRI scans in each session: one fMRI scan (under pain condition) at the beginning (pre) and at the end (post). In both sessions, whether the participants received spinal anesthesia or not (control condition) in between the two scans, the interval between the two scans was around one hour. To have acquired data at two time points allowed us to examine the reliability of brain response to the pain task across different conditions, and also to control for possible ordering effects in the absence of intervention, the spinal anesthesia. At the end of the study, participants were monitored until they were fully recovered from the spinal anesthetic, with a full return of motor functions and diuresis, and were then allowed to go home.

### Participants

Twenty-two right handed healthy male volunteers (aged 21–23 years) participated in the study after written informed consent was obtained and after approval of the protocol was given by the human ethics committee of the Leiden University Medical Center. The study was registered in the trial register of the Dutch Cochrane Center under identification number 3874. The study was performed according to Good Clinical Practice guidelines and the ethical principles of the Declaration of Helsinki (amended in 2013). All subjects were screened before participation in the study. Exclusion criteria included: body mass index >30 kg/m^2^; significant history or presence of any medical disorder, including bleeding disorders, or any medical issue that might interfere with optimal participation or pose any risk from spinal anesthesia; history of chronic alcohol or illicit drug use; the presence of metal devices; claustrophobia; allergy to study medications; and inability to maintain a regular diurnal rhythm.

### Procedures

**Figure 1:**
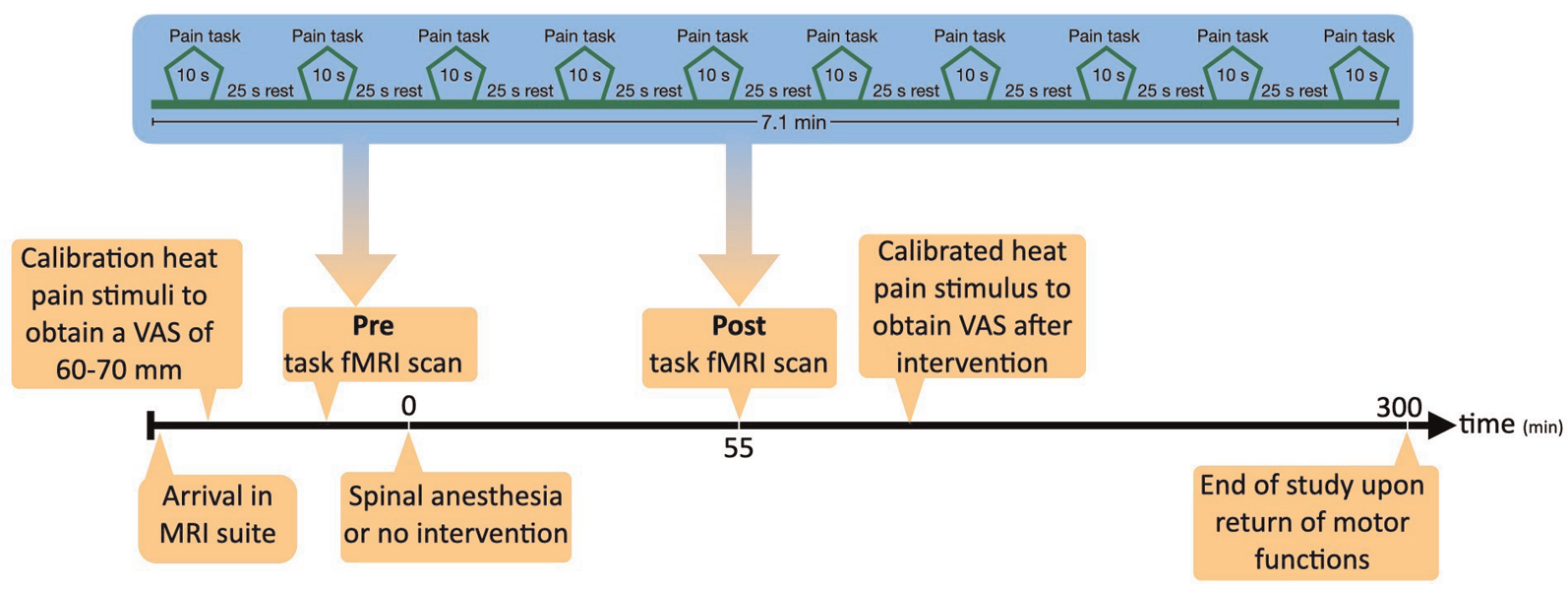
The timeline of the experiment.

Figure 1 depicts the timeline of the study procedures. After arrival in the MRI suite, a video demonstration of the spinal procedure was presented to the participant after which an intravenous access-line was placed in the left arm to allow administration of emergency medication if deemed necessary. After a short relaxation period, we calibrated pain stimulus to individual tolerance levels. Baseline thermal stimuli were applied on the right forearm to determine the temperature evoking a pain score of 60–70 mm out of 100 mm on a visual analog scale (VAS) for later use in the MRI experiment (see “pain task in the MRI scanner” below). This procedure was repeated during the second visit. The temperature used in the experiment could differ between the two visits, evoking the same heat pain score.

During the spinal anesthesia session, participants received an intrathecal injection with 15 mg bupivacaine (3 ml; AstraZeneca, Zoetermeer, the Netherlands) between vertebrae L3 and L4 (spinal condition). During the control session no injection was made.

### Noxious stimulus and interventions

Each session consisted of two task-based fMRI acquisitions (pre-scan and post-scan), one hour apart–duration necessary for administering and stabilization of the spinal anesthesia. During the wait interlude, ambulatory variables (blood pressure, heart rate and oxygen saturation) were monitored.

The noxious thermal stimuli (whose intensity was calibrated prior to the scan) were applied on the lower part of the right forearm with an MRI compatible 3 × 3 cm thermal probe attached to a Pathway Neurosensory Analyzer (Medoc Ltd., Ramat Yishai, Israel). During calibration and during the scans (pre and post) the temperature of the probe started at 32 °C (baseline temperature) and rapidly increased (5 °C/s) towards a preset destination temperature that was held constant for 10 s and then returned (5 °C/s) to baseline temperature. The heat pain stimulus was alternated with a 25 s lag time (block design). In total 10 pain stimuli were given, with a total task duration of 7.1 min (Figure 1).

The second MRI-scan (post) was conducted under the same stimulus intensity conditions as in in the first MRI-scan (pre). At the end of the post-scan, the noxious thermal stimuli were applied once more on the right forearm to measure VAS scores at the end of the scanning session. This procedure was necessary because we did not want to rate the pain during scanning to avoid confounds associated with cognitive processing of pain scores.

### Data acquisition

#### Scanning

Imaging data were acquired on a Philips 3 Tesla Achieva TX MRI scanner using a 32-channel SENSE head coil (Philips Medical Systems, Best, The Netherlands). Whole-brain fMRI data sets were acquired using T2*-weighted gradient-echo echo-planar imaging with the following scan parameters: 190 volumes; 38 axial slices scanned in ascending order; repetition time (TR) = 2.2 s; echo time (TE) = 30 ms; flip angle = 80˚; FOV = 220 × 220 mm; 2.75 mm isotropic voxels with a 0.275 mm slice gap. For registration purposes, a high-resolution anatomical image (T1-weighted ultra-fast gradient-echo acquisition; TR = 9.76 ms; TE = 4.59 ms; flip angle = 8°; 140 axial slices; FOV = 224 × 177.33 mm; in plane voxel resolution = 0.875 mm × 0.875 mm; slice thickness = 1.2 mm) was acquired for each participant. In order to control for confounding effects of experiment induced variations in physiological signals, participants were fit with a respiration belt and pulse oximeter, and for each fMRI dataset, ambulatory signals were measured at 500 Hz frequency.

#### fMRI data preprocessing

All fMRI scans were visually inspected to ensure that no gross artefacts were present. We excluded 4 datasets due to the presence of movement artefacts in the raw data, which could not be reliably removed or corrected (n = 18). Data preparation for fMRI included standard fMRI preprocessing, as well as additional cautionary physiological noise screening. For the standard fMRI preprocessing, FEAT (FMRI Expert Analysis Tool) Version 6.00, part of FSL (FMRIB's Software Library, www.fmrib.ox.ac.uk/fsl) was used with standard motion correction using MCFLIRT (3); skull removal using BET (4); and spatial smoothing (Gaussian kernel, 5 mm FWHM), as well as high pass temporal filtering (Gaussian-weighted least-squares straight line fitting, with sigma = 21.0s), and grand-mean intensity normalization of the entire 4D dataset by a single multiplicative factor.

Physiological noise monitoring was carried out according to (5). Briefly, this involved estimating the average heart rate (HR) (beats/min) and respiration variation (per min) obtained by taking the difference between respiration minimum and maximum values (peaks) divided by the time between the peaks and smoothed with a 6-s moving average filter. In addition, we performed RETROICOR (6) and RVHRCor (7) to the raw data (Using PhysIO (8) on CBRAIN (9). We subjected all physiological-corrected images to first-level statistics, to ensure that the activation patterns were not altered by noise.

#### First-level analysis of task-induced BOLD response

First level fMRI analysis was performed using FEAT (fMRI Expert Analysis Tool) Version 6.00. Time-series statistical analysis was carried out using FILM with local autocorrelation correction (10), including standard, and extended motion parameters in the subject level design matrix (block design, square shaped, including the temporal derivatives and temporal filtering). A standard double-Gamma hemodynamic response function was convolved with the box-car regressors in the model. We computed the Z-scores of the model fitting to the BOLD response in pain (on) versus no pain (off). In the first level analysis, positive BOLD responses (Z > 2.3) are reported as activation; and negative BOLD responses as deactivation.

#### Group analyses of the effects of interventions

Prior to group level analysis, we performed a multi-level image registration, by first registering each fMRI-dataset to a brain-extracted high-resolution T2*W image of the participant; then registering this data to the T1W image of each subject, and finally performing a registration to MNI152 (12 parameters).

Group Level Analysis was carried out using the high-level analysis feature in FEAT. Given the contextual specificity of the pharmacological intervention we used a fixed effects model, by forcing the random effects variance to zero in FLAME (FMRIB's Local Analysis of Mixed Effects) (11–13).

Different GLMs were constructed to test the effects of Time (post vs pre), Interventions (Spinal_(post−pre)_ versus Control_(post−pre)_), and stimulus intensity (temperature), and pain perception (VAS). In all models, the within-subject variations were modeled as an independent column per participant. In addition, because the trial was not blinded, we explored the effect of order on brain activation. To improve readability, we explained the details of the models in the results section.

All maps were thresholded at voxel-wise Z = 3.1, and cluster-corrected at *p* < 0.05.

### Statistical analysis

For studying the effects of stimulus intensity (temperature) and pain, we used SPSS 22, IBM. In order to investigate the impact of experimental conditions (condition, time and order of administering spinal anesthesia in the randomized design) on pain scores, we used generalized estimating equations (a specific form of generalized linear modeling that controls for within-subject variations in repeated measures studies such as ours.) Details are explained adjacent to the results.

## Results

### Effect of spinal anesthesia on pain intensity scores

The mean (± SEM) age of participants (18/22) was 21 ± 0,4 years, weight 73 ± 1,3 kg and height 183 ± 1,6 cm. The mean dermatome level of anesthesia during the first 50 min after spinal injection was at Th6 ± 3.5 (*i.e.,* at level of the xiphoid). The pain temperature, which induced a VAS of 60–70 mm, ranged from 44.5 to 50 °C (mean 48.3 ± 1.3 °C).

**Figure 2:**
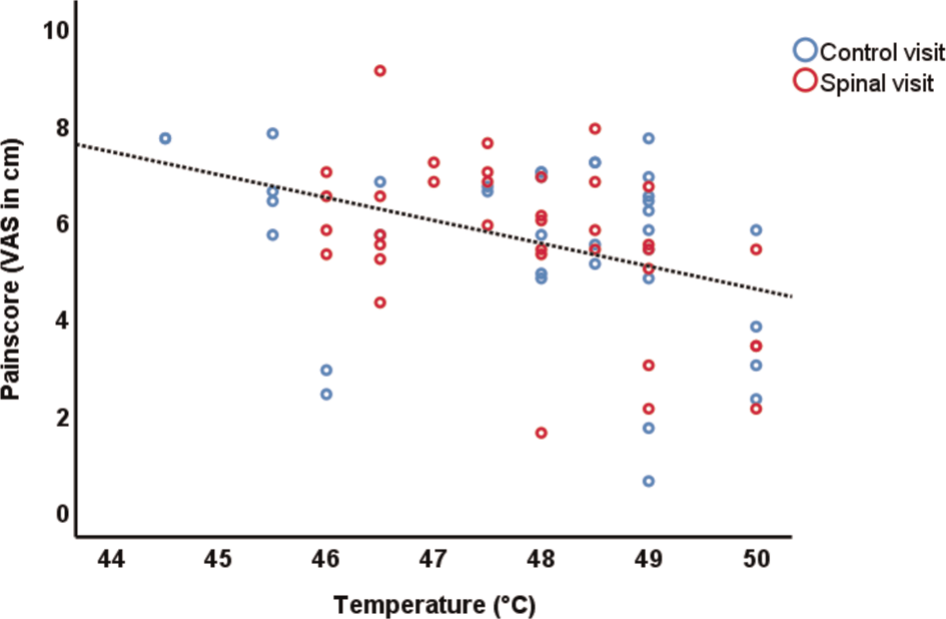
Scatterplot of the VAS scores and temperature of the control session and spinal anesthesia session.

We expected VAS scores and Temperature to be correlated. Indeed, performing partial correlation (controlling for Time and Session) we found a significant inverse correlation between VAS scores and temperature applied during the scan (*r* = −0.553, df = 68, *p* < 0.001), in other words, more than 30% of the variation in pain scores could be explained by the calibration temperature (Figure 2). The inverse correlation, surprising to us, might suggest that subjects who tolerated higher probe temperatures during the calibration session, had a lower pain sensitivity. As will be discussed later, the order of visits and receiving spinal anesthesia did impact the neural correlates of pain perception, however this correlation was significantly present in all conditions except in the control condition of participants who received spinal anesthesia in their first visit (See Supplementary Materials).

Table 1 summarizes the average pain scores at different time points in the study. A GEE model including Time, Session, Time by Session and Temperature, revealed significant time by session interaction effect on pain score (Wald *χ*^2^(df = 1)=7.73, *P* = 0.005), and this effect was mainly driven by higher pain scores after Spinal session (post-pre) compared to Control (post-pre) (95% CI = 0.233 to 1.345). It should be noted that the effect of session on temperature was not significant (Wald *χ*^2^(df = 1) = 0.54, *P* 0.46).

**Table 1:** Summary pain scores. Max VAS = 10 cm.

	Control session	Spinal session
	Mean ± SD	Mean ± SD
Mean temperature (°C)	47.86 ± 1.6	47.86 ± 1,27
Pre Pain VAS (cm)	5.61 ± 1.75	5.22 ± 1.77
Post Pain VAS (cm)	5.57 ± 1.98	5.97 ± 1.58

### BOLD response to noxious thermal stimuli

In order to investigate the replicability of the brain response to pain stimulation with respect to existing evidence from the Meta-analysis by Xu et al. (14), and to explore gross differences in the average response to spinal anesthesia compared to the control (non-anesthesia) condition, we tested the average effects of pain stimulation at each time point separately.

**Figure 3:**
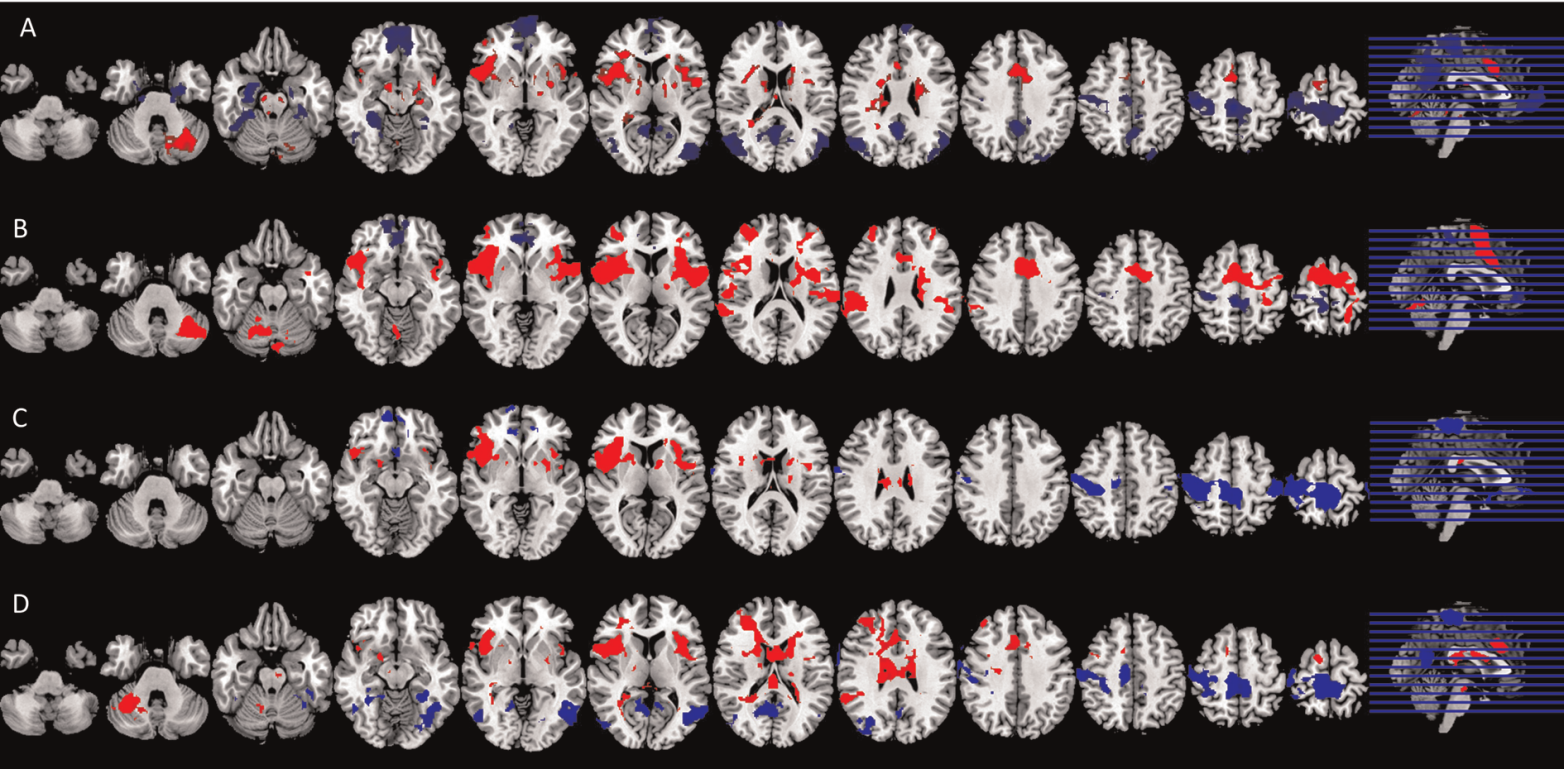
Significant neural responses to thermal noxious stimuli, resulting from a whole-brain analysis of (**A**) the control condition first scan (pre) and (**B**) the control condition second scan (post) (**C**) the spinal session first scan (pre) and (**D**) spinal session second scan (post). Significantly activated voxels are shown in red, significantly deactivated voxels in blue, *p* < 0.05 cluster corrected. Images are Z-statistics thresholded at (-)2.3, overlaid on the MNI-152 standard brain. Slices are displayed in radiological convention (left = right). All significant clusters are described in detail in Appendix Tables 1a, 1b, 2a, 2b.

Figure 3 shows average BOLD response to the noxious thermal stimulus for each MRI session. Table 2 summarizes the overlap between brain activations and deactivations in our study and the regions reported by Meta-analysis of Xu et al. (14) to be sensitive to sensation of pain.

**Table 2:** Brain region activation and deactivation per session and time (pre, post). Deactivation were not part of the meta-analysis of Xu et al. (14).

Brain activation upon thermal pain (Positive BOLD response)					
Location	Xu et al. (2020)	pre Control	post Control	pre Spinal	post Spinal
**Harvard-Oxford cortical structural atlas**					
Central opercular cortex	–	✓	✓	✓	✓
Frontal opercular cortex	–	✓	✓	✓	✓
Frontal pole	–	✓ (R)	✓	–	✓
Middle frontal gyrus	✓ (R)	–	–	–	✓ (R)
Insula	✓	✓	✓	✓	✓
Thalamus	✓	✓	✓ (L)	✓ (L)	✓
Anterior/ midcingulate gyrus	✓	✓	✓	–	✓
juxta positional cortex	–	✓ (R)	✓	–	✓
Brainstem	✓	✓	–	–	✓
Temporal pole	–	✓ (R)	✓	✓	✓
Cerebellum	✓ (L)	✓	✓	–	✓
Putamen	✓	✓	✓	✓	✓
Caudate	✓	✓	✓ (L)	✓	✓
Amygdala	✓	–	–	–	✓ (R)
Supramarginal gyrus	✓	–	–	–	✓ (R)
Precentral gyrus	✓ (R)	–	✓	–	✓ (R)
**Brain deactivation upon thermal pain (Negative BOLD response)**					
**Harvard-Oxford cortical structural atlas**					
Precentral gyrus		✓	✓	✓	✓
Postcentral gyrus		✓	✓	✓	✓
Frontal pole		✓	✓	✓	–
Frontal medial cortex		✓	✓	✓	–
Precuneus cortex		✓	–	–	✓
Cingulate gyrus, post. div.		✓	–	–	✓
Angular gyrus		✓	–	–	–
Lat. occipital cortex		✓	–	–	✓
Temporal fusiform cortex		✓	–	–	✓
Hippocampus		✓	–	–	–
Parahippocampal gyrus		✓	–	–	✓

Among the four conditions, we found generally comparable patterns of brain activation consistent with the literature. The noxious thermal stimuli caused consistent activations in the bilateral central opercular cortex, secondary somatosensory cortex, insula and thalamus. Consistent deactivations were present in all four scans in the precentral gyrus and postcentral gyrus.

However, the patterns of brain activation (red) and deactivation (blue) in the medial prefrontal and anterior cingulate regions appeared to be different in the case of spinal anesthesia (Figure 3D). Specifically, qualitative and quantitative inconsistencies were observed in activations in the anterior cingulate cortex, the brainstem, juxta positional lobule cortex, cerebellum, frontal operculum cortex, inferior frontal cortex and putamen, across the four scans. Similarly, deactivation across the four scans were inconsistent in the precuneus cortex, paracingulate gyrus, frontal medial cortex, middle/inferior temporal gyrus, lateral occipital cortex, hippocampus and angular gyrus. The frontal pole and medial frontal cortex were deactivated in all sessions, except in the post*-scan* during spinal anesthesia. See Figure 3, and Appendix Tables 1a, 1b, 2a, 2b for the complete lists.

### Effect of spinal anesthesia versus control

**Figure 4:**
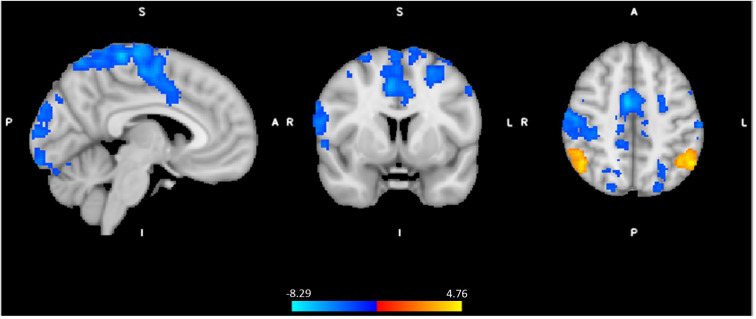
Significant neural responses to thermal noxious stimuli, resulting from a whole-brain analysis of the spinal session versus the control session (spin_(post−pre)_ - control _(post−pre)_), main effect of intervention. Red-yellow clusters correspond to higher BOLD response during spinal anesthesia compared to control, blue clusters correspond to a lower BOLD response during spinal anesthesia, *p* < 0.05 cluster corrected. Images are Z-statistics thresholded at (-)2.3, overlaid on the MNI-152 standard brain. A = Anterior, I = Inferior, L = Left, P = posterior, S = Superior, R = Right. The significant clusters are described in detail in Table 3.

In order to formally test the differences in brain activity during spinal anesthesia, we tested a general linear model (GLM) with all 72 data points, while modeling the random effect of subject, and fixed effects of time and intervention. In other words, the effect of time (Post-Pre), and the effect of condition (Spinal—Control) were treated as within subject variables. This model revealed significant differences in the BOLD response between the spinal anesthesia and control condition in the bilateral angular gyrus (IPL) (more positive) and the bilateral post- and precentral gyri (more negative) (Figure 4, Table 3).

**Table 3:** Spinal anesthesia effect on heat pain stimulus.

Spinal _(post-pre)_ > Control _(post-pre)_					
Location	Cluster size (1 mm^3^ voxels)	Peak Z-value	MNI coordinates (mm)		
			x	y	z
L angular gyrus	769	4.81	−52	−58	48
*This cluster also includes:					
L supramarginal gyrus		4.34	−54	−50	−40
R lateral occipital cortex	696	4.25	48	−60	52
*This cluster also includes:					
R angular gyrus		4.2	45	−56	52
**Spinal _(post-pre)_ < Control _(post-pre)_**					
R postcentral gyrus	19,742*	8.20	10	−46	76
*This cluster also includes:					
L precentral gyrus		7.97	−2	−34	68
L postcentral gyrus		7.15	−20	−40	72
R precentral gyrus		7.01	8	−18	78
L postcentral gyrus	642*	4.6	−60	−22	46
*This cluster also includes:					
L precentral gyrus		3.33	−54	6	42
L postcentral gyrus		3.3	−56	−10	32
L cerebral white matter		3.26	−38	−28	28

Significant clusters of brain response to spinal anesthesia (compared to control), significant at *p* < 0.05 cluster corrected. A Z-threshold of 2.3 was used; L = Left; R = Right.

**Figure 5:**
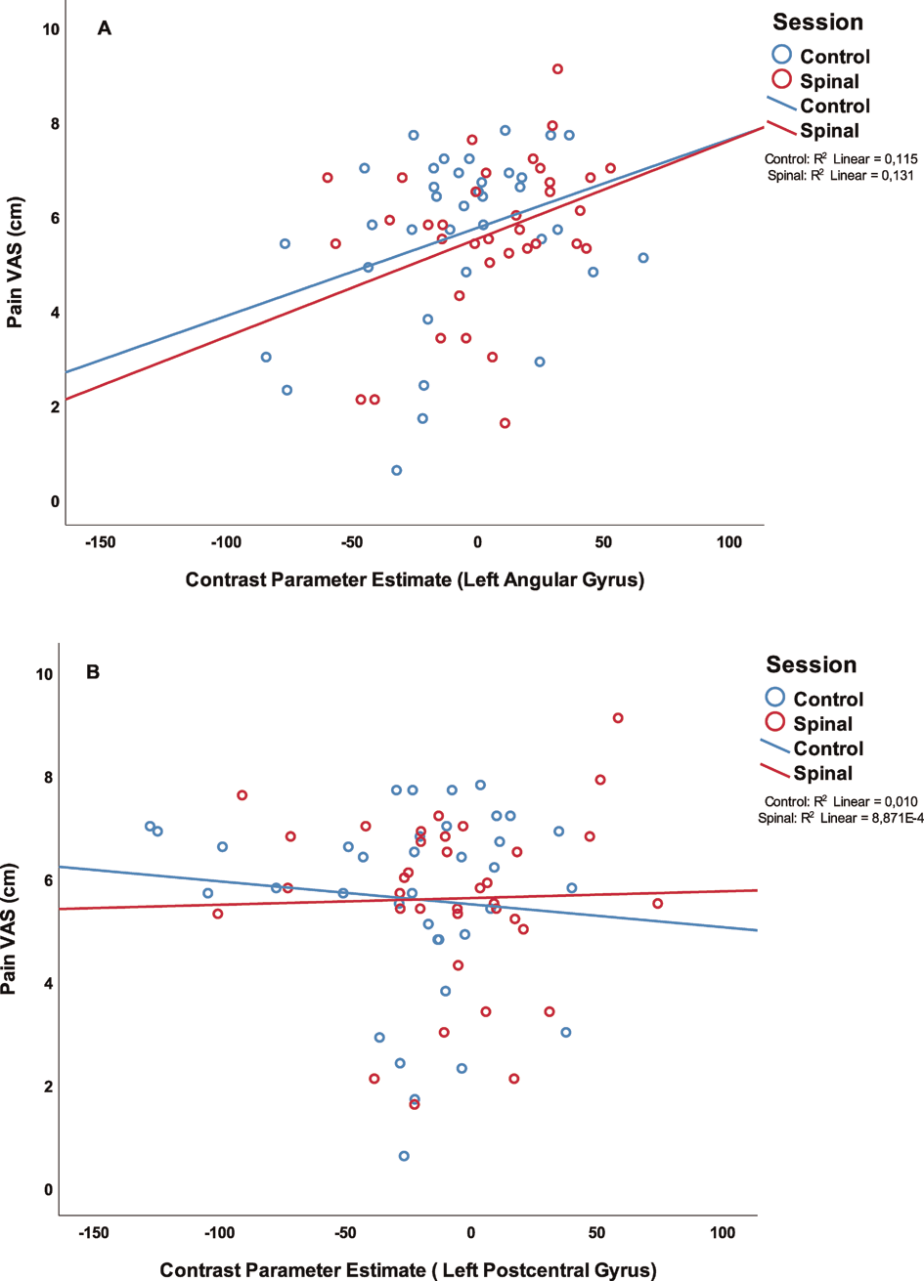
Scatterplot of the painscore (VAS in cm) and parameter estimates of (**A**): the peak voxel of the left angular gyrus, part of the IPL (X = −52, Y = −58, Z = 48 (**B**): the peak voxel of the left postcentral gyrus (X = −60, Y = −22, Z = 46). In blue the scores of the control session in red the scores of the spinal session.

In order to avoid circularity, we did not include effects of pain or temperature in the model described above. Instead, we asked whether brain regions activated by the task would be associated with pain perception. We extracted the contrast parameter estimates at the highest peak within the significant clusters where effects of spinal anesthesia (compared to control) was detected and performed a GEE by including Session × time × Pain score as predictive variables. This model revealed a significant interaction effect (Wald *χ*^2^(df =4) = 11.23, *P* = 0.024 on the peak activity in the inferior parietal lobule (IPL, X = −52, Y = −58, Z = 48). In all condition, except in the post scan during the control session, higher pain scores were significantly correlated with increased BOLD response in this region. A similar effect was also observed in the right side of the IPL (X = 60, Y = −48, Z = 30), (Wald *χ*^2^(df =4) = 13.39, *P* = 0.01), however correlations between pain score and BOLD activity were only significant in the post condition of the spinal session. Interestingly, we found no correlations between pain scores and peaks in the postcentral regions which seemed to be less activated during the spinal compared to control. However, testing a GEE with Temperature × Session by Time as a predictive variable showed a significant interaction effect (Wald *χ*^2^(df =4) = 9.73, *P* = 0.045) in the left postcentral region (X = 60, Y = −22, Z = 46), but no significant correlation across the four scans (P's > 0.08). Figure 5 illustrates the distribution of pain-scores and the contrast parameter estimate (COPE) at peaks. We used COPE instead of BOLD response percentage, because our stimulus was relatively long (∼10 s), and we did not have an existing estimate of the scale factor for a gamma-fit necessary to compute the percentage of the response.

### Effect of treatment order

**Figure 6:**
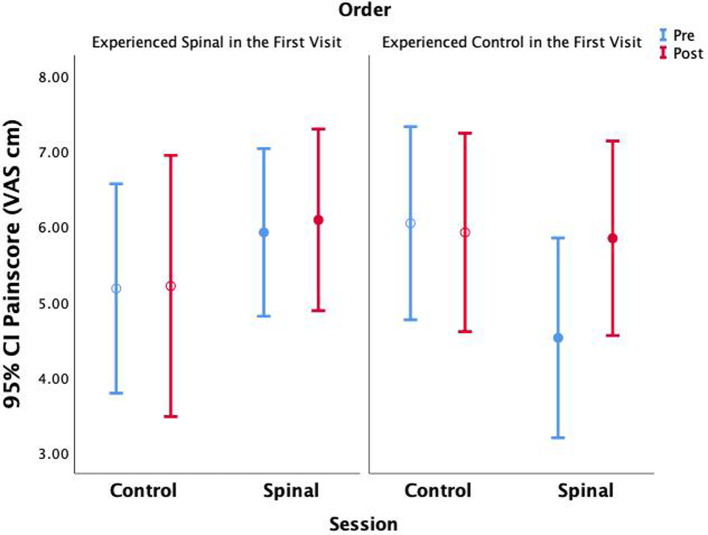
Average pain scores are shown without accounting for within subject parameter estimation across time and session.

Because the order of conditions was randomized, and the study could not be blinded, we examined whether the order of the two visits (spinal anaesthesia first visit or second visit) had any impact on pain perception. We repeated the GEE model by including order in the model (Pain VAS = Time × Session × Order + Time × Session + Time × Order + Session × Order + Time + Session + Order). This model revealed significant three-way interactions (Wald *χ*^2^(df = 1) = 7.58, *P* = 0.006), as well as two-way interaction between Time × Session (Wald *χ*^2^(df = 1) = 10.98, *P* = 0.001), thus confirming previous results; and two-way interaction between order × Session (Wald *χ*^2^(df = 1) = 7.01, *P* = 0.008). Compared to those who received the spinal session in the first visit, pain scores were lower in those who received the spinal anesthesia in the second visit (95% CI = −3.59 to −9.94). In those who received the spinal anesthesia in the second visit, the range of increase in pain perception was even more pronounced than what we found without accounting for the order effect (95% CI = 0.38 to 2.24) (Figure 6). However, the fact that this effect was missing in those who received the spinal anesthesia first might suggest that differences in anticipation of the intervention interacted with pain scoring. In the absence of more fine grained qualitative data, we will not be able to make inferences about this observation.

**Figure 7:**
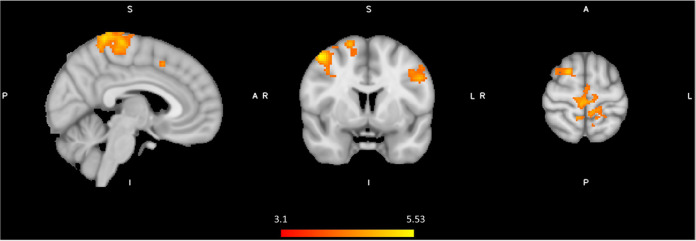
Significant neural responses to thermal noxious stimuli, resulting from a whole-brain analysis of the spinal condition visit first versus the spinal condition visit secondly (Spinal second_(post-pre)_ - Spinal first_(post-pre)_). Red clusters correspond to higher BOLD response during spinal anesthesia second visit compared to spinal anesthesia first visit, *p* < 0.05 cluster corrected. Images are Z-statistics thresholded at (-)3.1, overlaid on the MNI-152 standard brain. A = Anterior, I = Inferior, L = Left, P = posterior, S = Superior, R = Right. The significant clusters are described in detail in Table 4.

Figure 7 shows the results of the effect of order in the spinal condition visit on the BOLD response to noxious thermal stimuli. Those who experienced the spinal condition in the second visit, had significantly higher BOLD responses in several regions including the prefrontal and posterior areas. Between-conditions inconsistencies were also present namely, the bilateral precentral gyrus, right postcentral gyrus, left supramarginal gyrus, right lateral occipital cortex including precuneus cortex, right middle frontal gyrus including superior frontal gyrus (Figure 7, Table 4). The BOLD response in the angular regions [where significant spinal (versus control) effects were observed] were not affected by the order (P's > 0.4). Recall that although we had found an effect of spinal anaesthesia in the postcentral regions, they were not significantly associated with pain.

**Table 4:** Treatment order effect of spinal anaesthesia, spinal condition Second versus spinal condition First.

Spinal Second_(post-pre)_ > Spinal First _(post-pre)_					
Location	Cluster size (1 mm^3^ voxels)	Peak Z-value	MNI coordinates (mm)		
			x	y	z
R postcentral gyrus	1597*	5.28	8	−42	74
*This cluster also includes:					
R precentral gyrus		5.17	2	−26	70
L precentral gyrus		4.51	−8	−38	64
L supramarginal gyrus	1048*	4.26	−36	−50	32
*This cluster also includes:					
L angular gyrus		4.12	−46	−54	36
L lateral occipital cortex		3.94	−32	−68	38
R lateral occipital cortex	637*	3.95	28	−58	40
*This cluster also includes:					
R precuneus cortex		3.71	20	−64	38
R middle frontal gyrus	624*	5.48	46	4	56
*This cluster also includes:					
R superior frontal gyrus		4.49	20	8	64
R paracingulate gyrus		3.32	6	14	48
L precentral gyrus	441	4.19	−50	0	34

Significant clusters of brain response to spinal anesthesia (Second versus First) significant at *p* < 0.05 cluster corrected. A Z-threshold of 3.1 was used; L = Left; R = Right.

**Figure 8:**
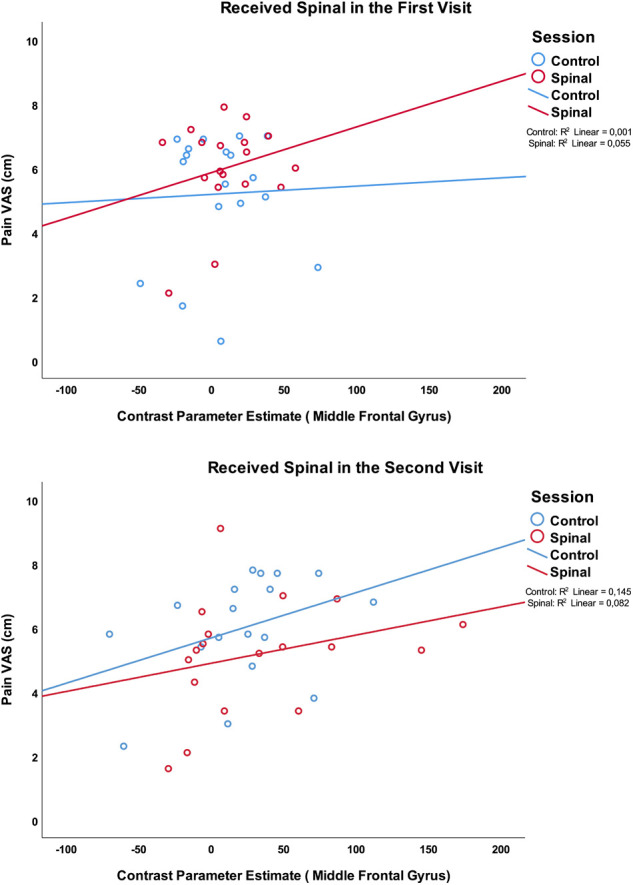
Association between pain scores and the medial prefrontal region which was significantly more activated in the group who received the spinal anesthesia in the second visit.

However, we observed a significant association between activity in the middle frontal gyrus (X = 46, Y = 4, Z = 56) and pain scores (GEE model variables: Pain score × Session × Time by order; Wald *χ*^2^(df =8) = 16.45, *P* = 0.036), with the effect being driven by positive correlation between BOLD response and pain in the control and the spinal conditions of those who received the Spinal anesthesia in the second visit. Figure 8 illustrates these correlations. It is noteworthy that while a consistent positive correlation is observed between pain scores and neural activity in three cases, this correlation was absent in the control condition of those who received spinal anesthesia on their first visit. This effect is similar to the observation of a discordant pain/temperature correlation during this condition, suggesting that other cognitive processes may have contributed to pain scoring during this presumably more “relaxed” condition (See Supplementary Materials).

## Discussion

We postulated that spinal anesthesia provides a plausible experimental model for studying pain associated with peripheral nerve damage. We tested this experimental model to replicate observations from our previous study, and to examine the effect of pharmacological spinal deafferentation on brain activation in response to a calibrated thermal pain stimulus. Indeed, we found a significant effect on pain sensitivity during deafferentation at the skin above the anesthetized dermatomes. The temporary deafferentation resulted in increased BOLD response to noxious thermal stimuli in the bilateral angular gyrus (IPL) and reduction of BOLD response in the pre and postcentral gyrus. Effects observed in the angular regions were associated with pain scores and were independent from order effect. We discuss the strength of our findings in the context of the existing body of knowledge and explain methodological challenges in mechanistic evaluations of the neurobiology of pain processing.

### Effect of noxious task on BOLD response

One of the strengths of our study is that we have acquired repeated task-based fMRI data under a common block-designed using thermal pain stimulation. This allowed us to examine the replicability of our results against the existing body of literature. Namely, as we have shown in Table 2, brain response to our fMRI task was to a large extent concordant with the findings of a recent meta-analysis of 222 fMRI studies of experimentally induced pain in healthy volunteers (14). In that study, Xu and colleagues found a core set of brain regions, including the thalamus, secondary somatosensory cortex (SII), insula and mid-cingulate cortex (MCC), to be activated irrespective of stimulus location or modality (14). For noxious thermal stimuli specifically, Xu and colleagues reported a subset of brain areas including the Rolandic operculum, MCC, middle frontal cortex, precentral gyrus and cerebellum to be activated. Our results also correspond to two other meta-analyses, except that they also emphasized the involvement of the anterior cingulate cortex (ACC) in response to pain (15, 16). Indeed, as can be seen in Figure 2, the ACC was involved in pain processing in all conditions, except pre-scan of the spinal session. As we will describe later, the absence of this effect may be due to anticipatory factors that resulted from random ordering of a condition that could not be blinded.

Our findings corroborate that a broad network is deactivated during exposure to noxious stimuli. The deactivated regions in our study are in line with deactivations reported by others (17–19). For example, Kong and colleagues have reported decreased activity in key regions of the default mode network (DMN), such as bilateral medial prefrontal cortex (MPFC), posterior cingulate cortex/precuneus, parahippocampus, hippocampus and lateral temporal cortex. They have also observed deactivation in brain regions involved in sensory motor analysis, such as lateral occipital gyri, premotor area, superior frontal gyrus, and ipsilateral primary S1/M1 (17). Often, reduced BOLD response in the first level analysis is assumed to represent a decrease in neuronal activity (20). We exercise caution in such interpretation, acknowledging the fact that the assumptions of a canonical hemodynamic response to a 10-seconds pain stimulus may not be valid.

Notwithstanding hemodynamic modeling limitations, the consistent deactivation in the ipsilateral sensorimotor cortex across the four conditions in our study is in line with the literature that attributes it to functional interhemispheric inhibition in order to optimize the differentiation of tactile information (21, 22). Transient suppression of the ipsilateral sensorimotor cortex during tactile finger stimulation using balloon diaphragms driven by compressed air has been described by Hlushchuk and colleagues (21). Both painful and sensory stimulation had the same transient suppression in ipsilateral sensory cortex, according to Taylor and colleagues (23).

The deactivation in the MPFC, known for its role in direction of internal conscious activity (24), in all but the post-scan in the spinal anesthesia visit, might suggest a disruption of the normal internally directed cognitive activity as a result of spinal anesthesia. This is not necessarily or directly related to pain processing. The experience of loss of sensation in the legs caused by the acute deafferentation could explain these differences, and be interpreted as an outcome of increased attention to a new experience, diverting attention from processing the pain stimulus. This interpretation is further supported by our observation of the proportionate reduction of brain activity in response to noxious stimuli, in the posterior part of the DMN (Figure 4).

### Effect of spinal anesthesia on brain activity

Spinal anesthesia causes a temporary paralysis of the lower part of the body and as such, it is a powerful model for disruption of afferent neural signaling from the limbs. Expectedly, pharmacological deafferentation is associated with changes in the network topography of brain regions involved in descending control and affective and sensory pain processing (2, 25, 26). In our previous resting-state fMRI study, we showed that spinal deafferentation was associated with increased pain sensitivity (hyperalgesia) (2); as well as a reduction in endogenous pain modulation expressed by reduced offset analgesia (27). A limitation of the previous study was that the noxious stimulus was administered outside the scanner. Therefore, performing a task-based fMRI under spinal anesthesia aimed to help us gain a more precise view into whether the previously observed changes were related to instantaneous pain processing.

In this current study, we observed that spinal anesthesia was associated with moderate hyperalgesia, as well as BOLD responses in the bilateral angular gyrus (IPL) that were significantly more activated during spinal anesthesia (compared to control). Desmurget and colleagues stimulated patients using a bipolar electrode during awake surgery at the inferior parietal regions, which resulted in intention to move with no actual movement. They concluded that the intention of motor movement emerges from the IPL bilaterally (28). Given that the angular gyrus is part of the IPL, which is important for mediation of movement intention and execution of motor tasks (28–31), we postulate that the observed effect reflects changes related to attentive pain processing.

The right-sided IPL is known to play a role in attention, encoding salient events and conflict tasks (32). The IPL is also part of multiple canonical resting state networks, such as the default mode network, the frontoparietal control network and the cingulo-opercular network (33, 34) that also constitute the affective pain network (35, 36). Given our study design, the highly localized difference observed in this region (during spinal anesthesia) corroborates interpretations by a previous study by Kong et al., that suggested IPL played a role in introspection and environmental monitoring (17). Budell and colleagues used healthy volunteers in a task-fMRI study with two tasks, one evaluating the amount of pain expressed (pain task) and the second discriminating movements (movement task) by watching one-second video clips displaying facial expressions of various levels of pain. They concluded that the bilateral IPL is predominantly involved in motor mirroring (31). Buckner and colleagues have suggested that the IPL serves as a communication hub where numerous networks converge and interact (37). Spinal anesthesia creates a strong somatosensory and interoceptive response as a result of losing sensation and the inability to move legs. We postulate that the effect observed by contrasting spinal versus control condition, irrespective of the order, was related to feeling the strong effect of this intervention.

Spinal anesthesia was also associated with reduced BOLD signal in the bilateral precentral and postcentral gyri. The postcentral gyrus receives somesthetic information of the body (38), partly blocked by the spinal anesthesia. Interestingly, effects in this region were not significantly correlated with pain scores, but an association with the temperature of the noxious stimuli was observed. Recall that we noted an inverse correlation between the thermal intensity and the pain score, suggesting that 30% of variations in pain perception were explained by pain tolerance. Moulton and colleagues differentiated heat sensation and pain sensation motivated by the fundamental concept that physical stimuli elicit distinguishable sensations such as heat besides pain. They concluded that the primary somatosensory cortex, positioned in the postcentral gyrus, better reflects the magnitude of heat sensation than pain intensity in experimental heat pain studies (39). Therefore, our results add to existing evidence for the involvement of these brain structures in conscious processing of sensorimotor-related activity, which were disrupted by a very strong somatosensory intervention.

### The effect of order of experiencing spinal anesthesia or control

One of the challenges in pharmacological neuroimaging is blinding. Randomization is a standard clinical practice that aims to remove the perceptual variations and reveal the mechanistic variations targeted by drugs. In the case of a complex sensation like pain, and a relatively complex and plausible stressful intervention, it is practically impossible to control for or remove all confounding effects. Although the increase in pain sensitivity caused by the spinal anesthesia (post—pre) seemed to be robust, we did observe a significant Session by Order interaction effect on pain scores (Figure 6), suggesting that anticipation may have played a role in pain scoring. The effect of ordering on main results observed in angular regions was not significant (P's > 0.4). However, comparing the effect of spinal anesthesia (versus control) in those who received the spinal intervention in the first visit versus those who received it in the second visit, revealed significant differences within the same regions where inconsistencies between the four conditions were observed.

We postulate that those who received the spinal anesthesia in the first visit were potentially more “relaxed” about what to expect in the second visit (no spinal anesthesia). By contrast, those who received the spinal anesthesia in the second visit were likely more hypervigilant about what to expect even during the control session. This interpretation is partially supported by the observation that the correlation between pain and temperature (See Supplementary Materials), were similar in all but those who experiences the control session in the second visit. Anticipation of upcoming events and experiences has been shown to impact the brain network that was activated by the noxious stimulation in our experiment (40). Indeed, we found that the BOLD response to the noxious heat stimuli in the task in those who received the spinal anesthesia in the second visit was higher in the right middle frontal gyrus and in the left superior frontal gyrus. As one of our reviewers suggested, the observed order effect could be due to the drug effects on pain reporting during the visit and not necessarily anticipation of pain itself (e.g., cognitive influences of pain processing). It is plausible to speculate that emotional and attentional differences caused by ordering might have contributed to the increase in pain scores (41–43). Activity in prefrontal and precentral regions are also reported to be associated with placebo-analgesia (44). We observed increased pain scores to be associated with the increased activity in the lateral prefrontal brain regions, during spinal anesthesia. The prefrontal cortex is known for its role in cognitive control of painful experiences (45–47). Because the examiners placed the heat probe on participant's arm, this lack of control may have contributed to stress as well. The prefrontal cortex is often reported to play and important role in cognitive stress modulation (48–50). Geva and colleagues examined the impact of an experimental psychosocial stress that modulates the prefrontal activity (50) on different dimensions of pain and showed that acute stress did not appear to impair pain sensitivity, but it did modulate the perception of pain magnitude, albeit with considerable interindividual differences (51). However, both increased and decreased pain sensitivity has been reported in presence of experimentally induced stress (52–54), which is plausibly related to interaction between different pain processing networks (55, 56), and the dynamics of their response to different sensory or affective factors (57). These issues need to be examined in a follow-up study with a more granular recording of perceptual and contextual experiences of study participants.

### Study limitations

Besides limitations in data collection to help us resolve the unexpected ordering effects, studies such as this are limited in blinding. Spinal anesthesia results in acute loss of motor and sensorimotor function of the lower part of the body, and the participants must be fully briefed about the procedure and expected effects prior to joining the study. Given the loss of motor and sensory control in the lower part of the body, those who received the intervention in the first session would be aware of what to not expect in the second session. Having observed the order effects, it is important to capture data that helps decipher the impact of acute unpleasantness, anxiety, or diversion during spinal anesthesia in the two sessions (spinal anesthesia and control).

Our study did not include any female participants. To include only male participants in pharmacological experiments is customary, as sex hormones influence pain sensitivity, especially during different phases of women's cycle (58). While this design reflects a pragmatic necessity (because otherwise, a larger sample is needed to control for hormonal cycles), interpretations from such designs remain very limited. Besides biological factors, gender differences in pain reporting might have psychosocial underpinnings (59). These differences may also manifest in cognitive and emotional processing of the noxious stimuli which will modulate neuronal activation (60, 61). To repeat this experiment in a sample including women is necessary.

We used standard first-level analysis using canonical hemodynamic response functions available in the FEAT (FSL V6.0). Some infrared spectroscopy imaging studies have shown that pain stimuli invoke a specific hemodynamic response (62, 63). In addition, the duration of the pain block (∼10 s) and differences in pain intensity (which was calibrated) might lead to hemodynamic response functions that are not fully captured with our canonical models (64, 65). We have explored some of these issues by introducing variations to the HRF model used for the first level analysis (e.g., by using different canonical functions, modeling the effect of temperature and time of stimulus onset, as well as removal of respiratory and cardiac pulses, in the first level analysis), however, we did not observe differences in the topography of first order effects. Future studies need to explore the impact of block duration, as well as event-related pain stimulus tasks on results.

*In conclusion,* the loss of sensory and motor activity caused by spinal anesthesia has a significant impact on brain regions involved in the sensorimotor and cognitive processing of noxious thermal stimuli. Alterations in pain sensitivity were seen in non-deafferented skin regions, i.e., at dermatomes above the level of the spinal anesthetic in a subset of participants. Treatment order significantly influenced pain sensitivity and activation of brain regions involved in heat sensation and cognitive processing of pain. This important and unexpected observation warrant attention in design of future randomized controlled trials that cannot be blinded. In these cases, additional psychometric, and phenomenological data can improve interpretations.

## Data Availability

The raw data supporting the conclusions of this article will be made available by the authors, without undue reservation.

## Appendix

**Appendix Tables 1a:** Brain activation and deactivation pre scan control condition.

Brain activation upon thermal pain					
Location	Cluster size (1 mm^3^ voxels)	Peak Z-value	MNI coordinates (mm)		
			x	y	z
R temporal pole	7398*	4.21	56	10	−2
*This cluster also includes:					
R Anterior cingulate gyrus		4.21	0	12	40
R Central opercular cortex		4.2	52	6	0
R juxta positional cortex		4.14	12	4	54
brainstem		4.04	10	−18	−20
L anterior cingulate gyrus		3.89	−10	6	34
B cerebellum	927	3.95	−32	−60	−30
**Brain deactivation upon thermal pain**					
R precentral gyrus	4075*	4.03	4	−28	60
*This cluster also includes:					
R postcentral gyrus		3.68	48	−24	60
B frontal pole	2498*	3.78	0	70	12
*This cluster also includes:					
R frontal medial cortex		3.59	12	40	−12
R precuneus cortex	2283*	3.79	6	−54	10
*This cluster also includes:					
R cingulate gyrus, post. div.		3.66	2	−52	30
L angular gyrus	1762*	3.99	−52	−60	24
*This cluster also includes:					
L lat. occipital cortex		3.63	−40	−72	10
R lat. occipital cortex	1420	3.77	52	−68	26
R temporal fusiform cortex	1408*	3.86	24	−36	−22
*This cluster also includes:					
R hippocampus		3.73	30	−10	−22
R parahippocampal gyrus		3.58	24	0	−22
L parahippocampal gyrus	700	3.71	−30	−26	−24

Brain activation and deactivation in response to thermal pain, significant at *p* < 0.05 cluster corrected. A Z threshold of 2.3 was used. B = Bilateral; L = Left; R = Right.

**Appendix Tables 1b:** Brain activation and deactivation post scan control condition.

Brain activation upon thermal pain					
Location	Cluster size (1 mm^3^ voxels)	Peak Z-value	MNI coordinates (mm)		
			x	y	z
L central opercular cortex	9500*	5.47	−50	2	6
*This cluster also includes:					
L insular cortex		5.39	−36	4	8
Juxta positional lobule cortex		4.9	2	−4	68
R insular cortex	5990*	5.37	46	6	−2
*This cluster also includes:					
R Central opercular cortex		5.32	50	2	8
R temporal pole		5.32	52	8	−4
Cerebellum	1840	4.5	−40	−64	−30
**Brain deactivation upon thermal pain**					
R postcentral gyrus	1340*	4.4	16	−30	76
*This cluster also includes:					
R precentral gyrus		3.56	30	−26	56
L precentral gyrus		3.37	−2	−34	62
R cingulate gyrus, anterior div.	1256*	3.78	12	42	0
*This cluster also includes:					
R paracingulate cortex		3.74	4	42	−4
R frontal pole		3.23	16	58	−10
L subcallosal cortex		3.06	−6	28	−8

Brain activation and deactivation in response to thermal pain, significant at *p* < 0.05 cluster corrected. A Z-threshold of 2.3 was used. B = Bilateral; L = Left; R = Right.

**Appendix Tables 2a:** Brain activation and deactivation pre scan spinal condition.

Brain activation upon thermal pain					
Location	Cluster size (1 mm^3^ voxels)	Peak Z-value	MNI coordinates (mm)		
			x	y	z
R central opercular cortex	3009*	4.48	50	0	8
*This cluster also includes:					
R frontal operculum cortex		4.27	38	16	6
R insular cortex		4.16	40	18	0
R inferior frontal gyrus		3.91	48	14	12
L central opercular cortex	1791*	4.13	−38	2	12
*This cluster also includes:					
L frontal opercular cortex		4.02	−36	16	6
L WM sup. occipito-frontal fascicle		3.87	−20	4	22
L putamen		3.64	−28	0	−6
**Brain deactivation upon thermal pain**					
R postcentral gyrus	5240*	4.67	56	−12	54
*This cluster also includes:					
R precentral gyrus		4.05	30	−22	66
L paracingulate gyrus	835*	3.74	−10	38	−8
*This cluster also includes:					
R frontal medial cortex		3.35	4	46	−10
R paracingulate gyrus		3.35	4	40	−6
Subcallosal cortex		3.27	−2	14	−14
L postcentral gyrus	635	3.46	−54	−22	60

Brain activation and deactivation in response to thermal pain, significant at *p* < 0.05 cluster corrected. A Z threshold of 2.3 was used. B = Bilateral; L = Left; R = Right.

**Table 4b:** Brain activation and deactivation post scan spinal condition.

Brain activation upon thermal pain					
Location	Cluster size (1 mm^3^ voxels)	Peak Z-value	MNI coordinates (mm)		
			x	y	z
R paracingulate gyrus	9265*	4.36	2	24	34
*This cluster also includes:					
L central opercular cortex		4.24	−38	8	8
L WM		4.21	−8	−4	24
R central opercular cortex		4.15	40	10	4
L insular cortex		4.11	−38	14	2
R cerebellum	619	3.71	38	−56	−30
**Brain deactivation upon thermal pain**					
R postcentral gyrus	4875	4.67	2	−38	66
L lat. Occipital cortex	2075*	4.13	−58	−64	6
*This cluster also includes:					
L temporal occipital fusiform cortex		3.65	−38	−58	−14
L middle temporal gyrus		3.39	−56	−54	0
R lingual gyrus	1449*	3.46	18	−42	−16
*This cluster also includes:					
R precuneus cortex		3.43	14	−50	10
R lateral occipital cortex	848*	3.28	32	−72	24
*This cluster also includes:					
R inferior temporal gyrus		3.16	48	−56	−12

Brain activation and deactivation in response to thermal pain, significant at *p* < 0.05 cluster corrected. A Z threshold of 2.3 was used. B = Bilateral; L = Left; R = Right.
