# Supplementary material for: Effect of spinal anesthesia-induced deafferentation on pain processing in healthy male volunteers: A task-related fMRI study
Source: Front Pain Res (Lausanne). 2022 Nov 30;3:1001148. doi: 10.3389/fpain.2022.1001148 (PMC9748364; doi:10.3389/fpain.2022.1001148)
Supplement: Supplementary file 1 [file Datasheet1.pdf]

Supplementary Materials

Correlation between pain scores and temperature are plotted by splitting the sample according to the order of the visit. This shows a consistent inverse correlation in both spinal and control condition of those who received Control in the first visit and Spinal in the second visit. However, this correlation was absent in cases where participants received the control session in their second visit.

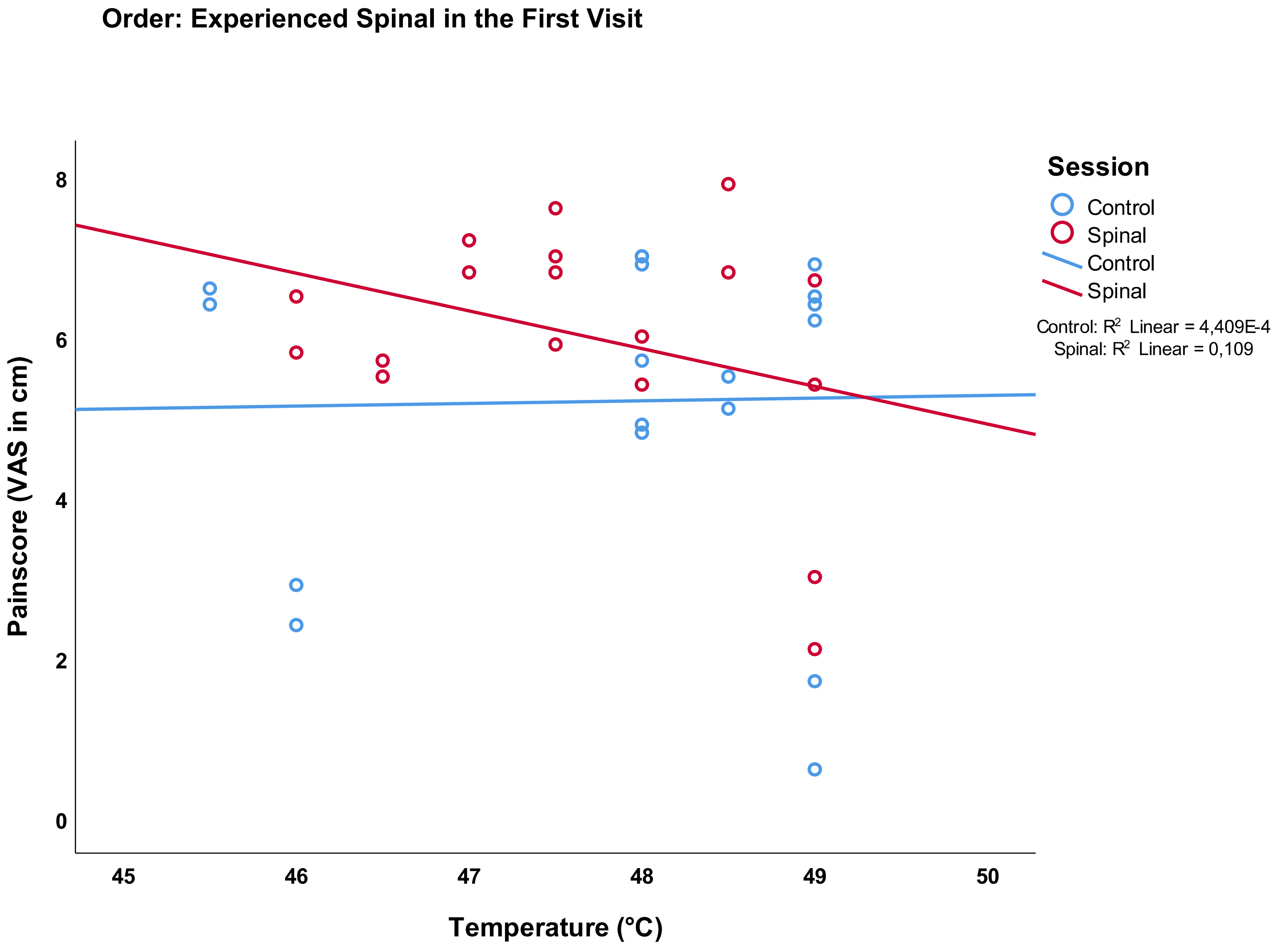

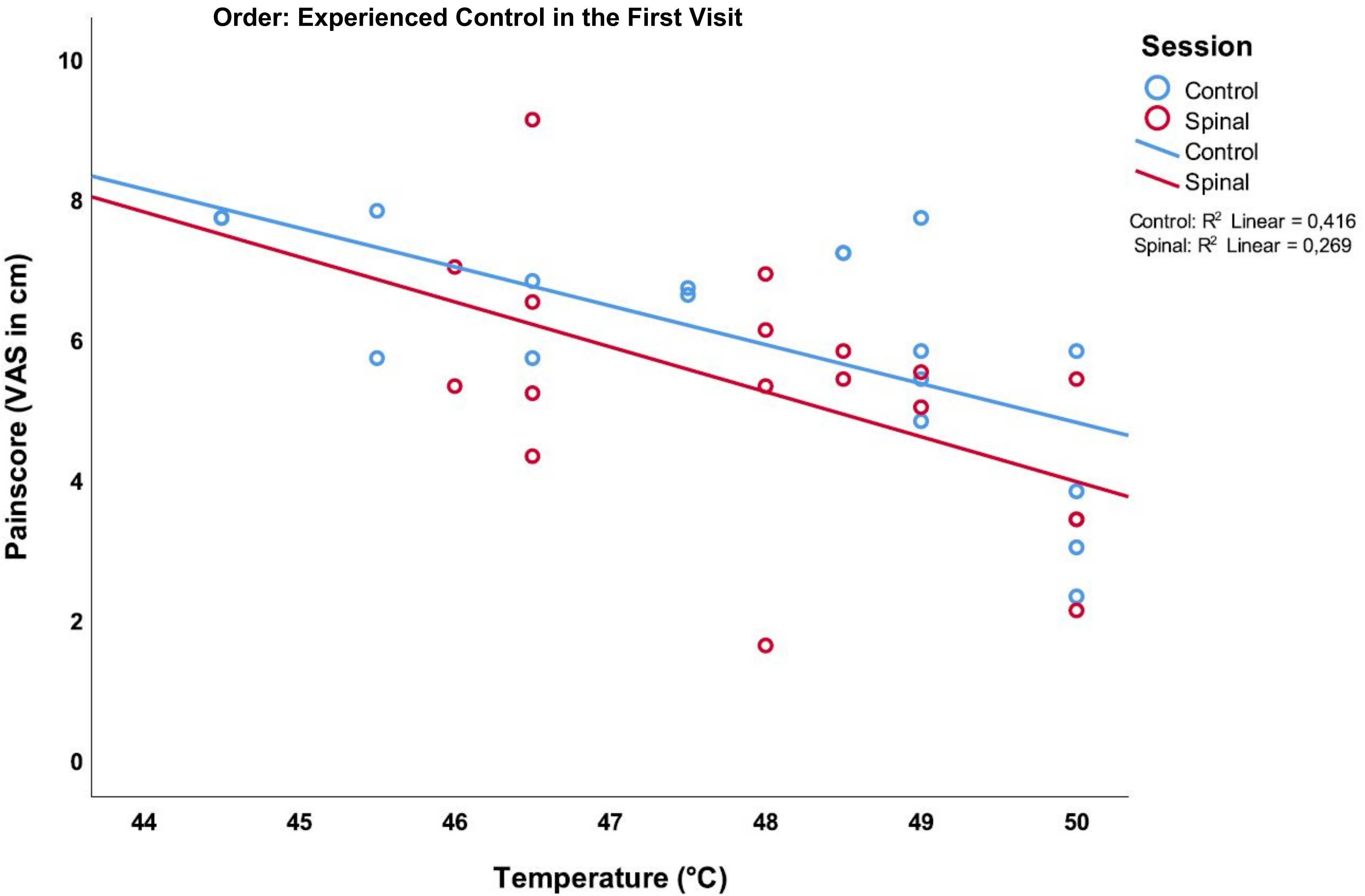
Order: Experienced Control in the First Visit
Session
Control
Spinal
Control
Spinal
Control: R2 Linear = 0,416
Spinal: R2 Linear = 0,269
Painscore (VAS in cm)
10
8
6
4
2
0
44
45
46
47
48
49
50
Temperature (°C)
